# Increased Levels of Cardiac Troponin I in Subjects with Extremely Low B-type Natriuretic Peptide Levels

**DOI:** 10.1038/s41598-018-23441-z

**Published:** 2018-03-23

**Authors:** Satoshi Sugawa, Izuru Masuda, Kiminori Kato, Michihiro Yoshimura

**Affiliations:** 1 Diagnostics Division, Abbott Japan Co., Ltd, Tokyo, Japan; 2 Takeda Hospital Medical Examination Center, Kyoto, Japan; 3 Niigata Medical Association of Occupational Health, Inc, Niigata, Japan; 4 Division of Cardiology, Department of Internal Medicine, The Jikei University School of Medicine, Tokyo, Japan

## Abstract

Because of the lack of studies focused on the biological implications of extremely low B-type natriuretic peptide (BNP) levels, we investigated whether extremely low BNP levels could be harmful to the cardiovascular system due to compromised cardio-protection. By using cardiac troponin I (cTnI) as an indicator of cardiovascular disorder, we assessed whether cTnI was inversely associated with BNP in populations with low BNP levels. A total of 2,001 apparently healthy subjects older than 38 years were included in this study. We defined subgroups from this population by limiting the maximum BNP level with cut-off values ranging from 1 through 20 pg/mL and performed covariance structure analyses by comparing log(BNP) with log(cTnI) in each subgroup. The beta values between log(BNP) and log(cTnI) sharply decreased as the BNP cut-off was reduced from 20 pg/mL (beta = 0.04) to 1 pg/mL (beta = −0.29) and became significant when the BNP cut-off levels were lower than 4 pg/mL (p < 0.005). In subgroups with BNP levels lower than 4 pg/mL, elevation in cTnI level was inversely associated with BNP (p < 0.005), which suggests that insufficient BNP may play a pathogenic role in the occurrence of cardiovascular abnormalities.

## Introduction

B-type natriuretic peptide (BNP), a peptide that was discovered by Sudoh *et al*.^1^, is predominantly expressed in ventricles and secreted in response to several factors, including myocardial stretching, increased myocardial pressure, and cell hypoxia, to bring about pharmacological effects, such as vasorelaxation, diuresis, natriuresis, and the inhibition of the renin-angiotensin-aldosterone system^2, 3^. As BNP elevation is correlated with the severity of cardiac dysfunction in patients with heart failure, BNP is used for the diagnosis, stratification and monitoring of heart failure in clinical settings^4^. BNP has also been demonstrated to be correlated with the Framingham Risk Score, which indicates its usefulness for assessing the risk of coronary heart disease in the general population^5^.

The BNP cut-off level was reported at 18.4 pg/mL in 1993^6^, but 40 pg/mL was proposed in 2012 as an alternative cut-off for practical use in clinical settings^7^. In either case, there is a basic understanding that lower BNP levels are associated with better prognoses. Among the numerous previous reports on BNP, we could not find any article that focused on the biological implications of extremely low BNP levels (e.g., BNP levels below 4 pg/mL).

Cardiac troponin I (cTnI) is expressed in the myocardium as a cardio-specific isoform, and thus it is ideally suited as a marker of myocardial damage^8^. Owing to the development of high-sensitivity troponin assays in recent years, the measurement of troponin levels with those assays for the diagnosis of acute coronary syndrome (ACS) is stipulated as more reliable than measurements with any other conventional biomarkers based on latest guidelines^9,10^. High-sensitivity troponin assays such as the cTnI assay used in this study are not only used for the diagnosis of ACS but also known to have the ability to predict cardiovascular events in the general population. By measuring cTnI levels with the high-sensitivity cTnI assay in a Scottish cohort consisting of 15,340 individuals from the general population, Zeller *et al*. showed that cTnI was associated with future cardiovascular events over an average of 20 years of follow-up and suggested threshold values of 4.7 pg/mL for women and 7.0 pg/mL for men to identify individuals at risk of future cardiovascular events^11^; these suggested threshold values are far below the 99^th^ percentiles of cTnI concentrations indicated in the package insert of the assay – 15.6 pg/mL for women and 34.2 pg/mL for men.

In a 10-year follow-up of the Inter99 study, Hansen *et al*. showed with a cohort of 6,238 general population that metabolically healthy obese subjects had a higher risk of ischaemic heart disease (IHD) than metabolically healthy subjects with normal weight^12^. As shown by several reports, BNP level is reduced in individuals with obesity independent of the presence or absence of cardiac diseases^13–15^. The metabolically healthy obese subjects in the Inter99 Study are therefore considered to be equivalent to individuals with reduced BNP levels. Because of the cardio-protective effects exerted by BNP, we hypothesized that the increased risk of IHD in individuals with obesity is due not only to well-known risk factors (e.g., hypertension, dyslipidaemia, diabetes) but also to the compromised cardio-protection of the reduced BNP level. By a covariance structure analysis in 1,252 patients with cardiac disorders, we confirmed that low BNP level, as well as hypertension, dyslipidaemia and haemoglobin A1c (HbA1c), but not body mass index (BMI) was significantly associated with the incidence of IHD (p < 0.001)^16^. To confirm this hypothesis further, by using cTnI as an indicator of cardiac disorders in this study, we assessed whether low BNP level was associated with an enhanced cardiovascular risk in the general population.

## Results

### Characteristics of the study population

The characteristics of the total cohort (884 females and 1,117 males) are presented in Table 1. Briefly, the median values of the age, BMI, BNP level and cTnI level of the subjects were 56.0 years, 22.6 kg/m^2^, 5.6 pg/mL and 1.5 pg/mL, respectively. The median Framingham Risk Score was 10.0, which meant a 6.3% 10-year cardiovascular risk for the women and a 9.4% risk for the men according to a previous report^17^.

**Table 1:** Basic Characteristics of the Total Cohort.

	Unit	N = 2,001 (884 females and 1,117 males)
		Median (25%ile, 75%ile)
Age	years	56.0 (48.0, 63.0)
BMI	kg/m^2^	22.6 (20.6, 24.8)
WC	kg/m^2^	81.5 (75.7, 87.5)
SBP	mmHg	119.0 (109.0, 129.0)
DBP	mmHg	76.0 (68.0, 84.0)
Heart Rate	bpm	67.0 (61.0, 75.0)
Vital Capacity	L	3.4 (2.8, 4.1)
WBC	/μL	5200.0 (4300.0, 6100.0)
RBC	10^4^/μL	462.0 (433.0, 491.0)
Hb	g/dL	14.2 (13.3, 15.2)
Ht	%	43.0 (40.5, 45.6)
PLT	10^4^/μL	23.6 (20.6, 27.3)
ALB	g/dL	4.4 (4.2, 4.6)
AST	U/L	21.0 (18.0, 25.0)
ALT	U/L	19.0 (14.0, 26.0)
GGT	U/L	26.0 (17.0, 45.0)
UA	mg/dL	5.2 (4.3, 6.2)
eGFR	mL/min/1.73 m^2^	75.3 (67.5, 84.8)
BUN	mg/dL	13.0 (11.0, 16.0)
LDL-C	mg/dL	124.0 (106.0, 144.0)
HDL-C	mg/dL	62.0 (51.0, 75.0)
TG	mg/dL	88.0 (62.0, 130.0)
HbA1c	% (NGSP)	5.6 (5.4, 5.9)
Fasting Blood Glucose	mg/dL	98.0 (92.0, 106.0)
Framingham Risk Score		10.0 (7.0, 14.0)
BNP	pg/mL	5.6 (3.0, 10.3)
cTnI	pg/mL	1.5 (0.6, 2.8)
Current smoker	%	35.6
CKD	%	10.4
Dyslipidaemia	%	65.3
Hypertension	%	33.4
Diabetes	%	32.2

BMI, body mass index; WC, waist circumference; SBP, systolic blood pressure; DBP, diastolic blood pressure; WBC, white blood cell count; RBC, red blood cell count; Hb, haemoglobin; Ht, haematocrit; PLT, platelet count; ALB, albumin; AST, aspartate aminotransferase; ALT, alanine aminotransferase; GGT, gamma glutamyl transferase; UA, uric acid; eGFR, estimated glomerular filtration rate; BUN, blood urea nitrogen; LDL-C, low-density lipoprotein cholesterol; HDL-C, high-density lipoprotein cholesterol; TG, triglycerides; HbA1c, haemoglobin A1c; BNP, B-type natriuretic peptide; cTnI, cardiac troponin I; CKD, chronic kidney disease.

### Analytical performance of the BNP assay

We performed a reproducibility test for the BNP assay used in this study and assessed the limit of quantitation (LoQ) as described in Materials and Methods. As shown in Table 2, the total error, defined as the sum of the coefficient of variation (CV%) and absolute bias, for each diluted sample with BNP levels ranging from 0.8 pg/mL to 54.2 pg/mL was within 30%. From this result, the LoQ of the BNP assay was determined to be 0.8 pg/mL.

**Table 2:** Evaluation of the Quantifiable Range of the BNP Assay.

N	Unit	Blank	Dilution factor						Cont. L
			64	32	16	8	4	2	
1	pg/mL	0.0	0.9	1.5	3.3	6.3	14.6	28.6	60.7
2	pg/mL	0.0	0.8	1.9	3.0	6.7	14.0	27.4	53.8
3	pg/mL	0.0	1.0	1.8	3.3	6.5	14.2	26.6	55.2
4	pg/mL	0.0	0.8	1.5	3.2	6.2	13.5	27.1	52.6
5	pg/mL	0.0	0.7	1.6	3.1	6.4	13.3	28.2	55.3
6	pg/mL	0.0	0.7	1.5	3.2	6.8	13.3	27.2	52.3
7	pg/mL	0.0	0.7	1.3	3.3	6.4	13.1	28.4	56.2
8	pg/mL	0.0	0.7	1.6	3.5	6.7	13.0	25.9	49.8
9	pg/mL	0.0	0.6	1.7	3.2	6.3	13.3	27.3	54.3
10	pg/mL	0.0	0.6	1.6	3.1	6.6	13.0	26.2	52.1
Expected value (mean)	pg/mL	0.0	0.8	1.7	3.4	6.8	13.6	27.1	54.2
Measured value (mean)	pg/mL	0.0	0.8	1.6	3.2	6.5	13.5	27.3	54.2
Bias (%)	%	—	−10.3	−4.4	−4.8	−4.3	−0.3	0.7	0.0
CV%	%	—	16.7	11.4	3.9	3.0	4.1	3.4	5.4
Abs(bias)% + CV%	%	—	27.0	15.8	8.8	7.3	4.4	4.0	5.4

The samples were prepared by diluting Control L by dilution factors signified on the top row. CV%, coefficient of variation; abs(bias), absolute value of the bias.

### Two-dimensional distribution of BNP and cTnI

**Figure 1:**
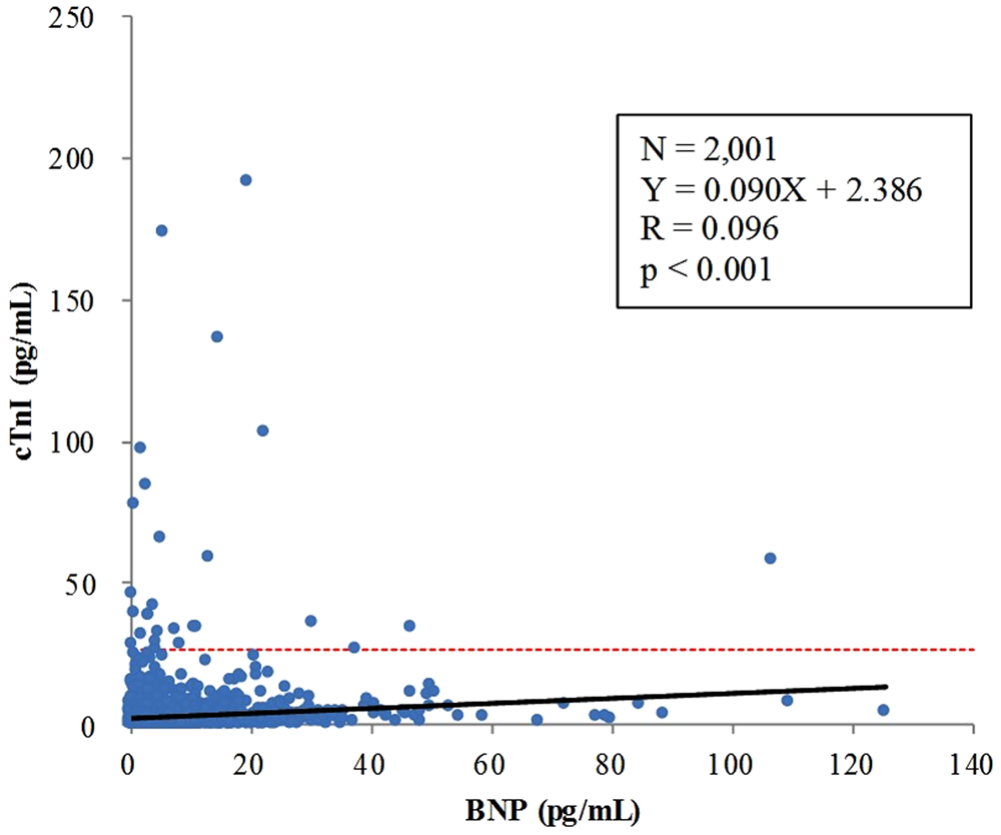
Two-dimsensional plot of BNP (X-axis) and cTnI (Y-axis) levels. The solid line () signifies linear regression. The red dotted line () signifies the cut-off level for cTnI at 26.2 pg/mL. BNP, B-type natriuretic peptide; cTnI, cardiac troponin I; WC, waist circumference; SBP, systolic blood pressure; eGFR, estimated glomerular filtration rate; HbA1c, haemoglobin A1c.

A two-dimensional distribution chart was constructed by plotting BNP level on the X-axis and cTnI level on the Y-axis for the total cohort (N = 2,001) as illustrated in Fig. 1. The correlation coefficient of the linear regression of the distribution was 0.096 (p < 0.001), and the regression formula was Y = 0.090X + 2.386.

### Metabolic derangement in the low BNP subgroups

**Figure 2:**
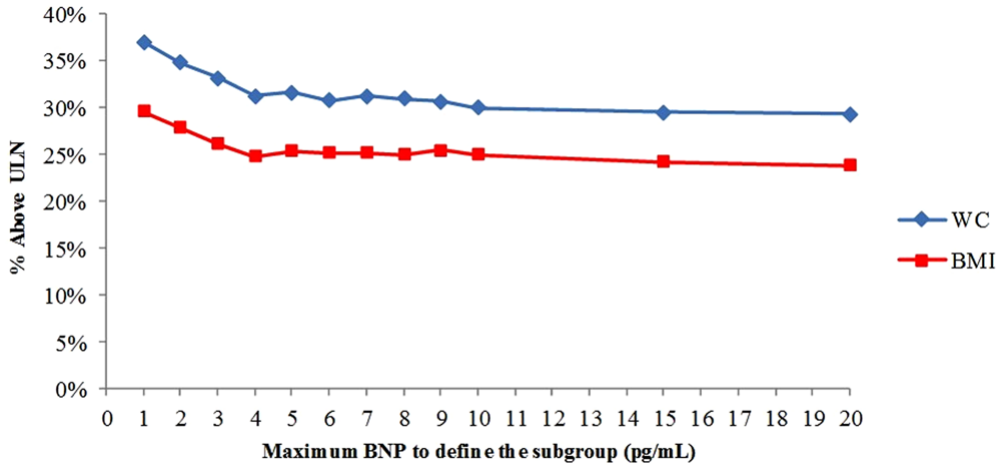
Percentage of subjects exceeding the ULN WC or BMI (Y-axis) in subgroups defined by maximum BNP levels (X-axis). BNP, B-type natriuretic peptide; WC, waist circumference; BMI, body mass index; ULN, upper limit of normal.

To evaluate the presence of metabolic derangement in patients with extremely low BNP levels, we determined the percentage of subjects who exceeded the upper limit of normal (ULN) WC or BMI. As shown in Fig. 2, drastic increases in the percentage of subjects exceeding the ULN WC and BMI were seen in subgroups with BNP levels lower than 4 pg/mL.

### Covariance structure analyses

**Figure 3:**
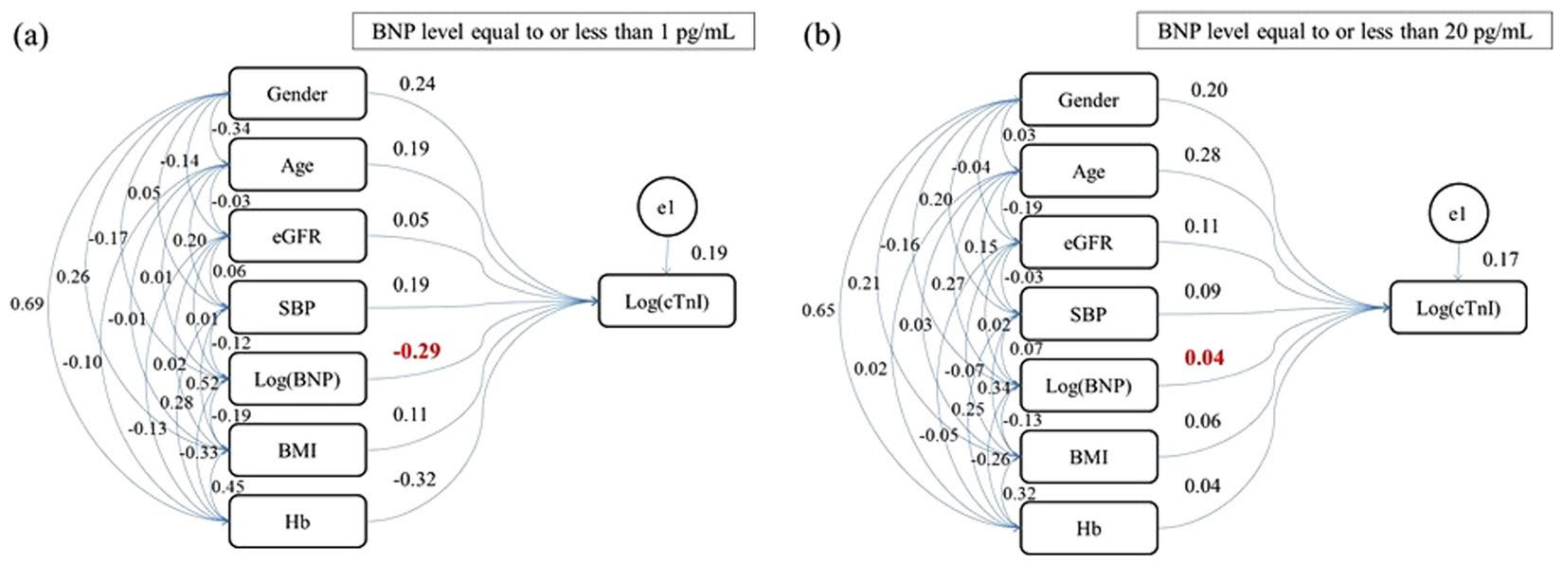
(**a**) Path diagram against log(cTnI) in subjects with BNP levels equal to or lower than 1 pg/mL (low BNP group, N = 81). (**b**) Path diagram against log(cTnI) for subjects with BNP levels equal to or lower than 20 pg/mL (control group, N = 1,860). The coefficient of standardized regressions (direct effects) of each path is shown next to the path. cTnI, cardiac troponin I; eGFR, estimated glomerular filtration rate; SBP, systolic blood pressure; BNP, B-type natriuretic peptide; BMI, body mass index; Hb, haemoglobin.

**Figure 4:**
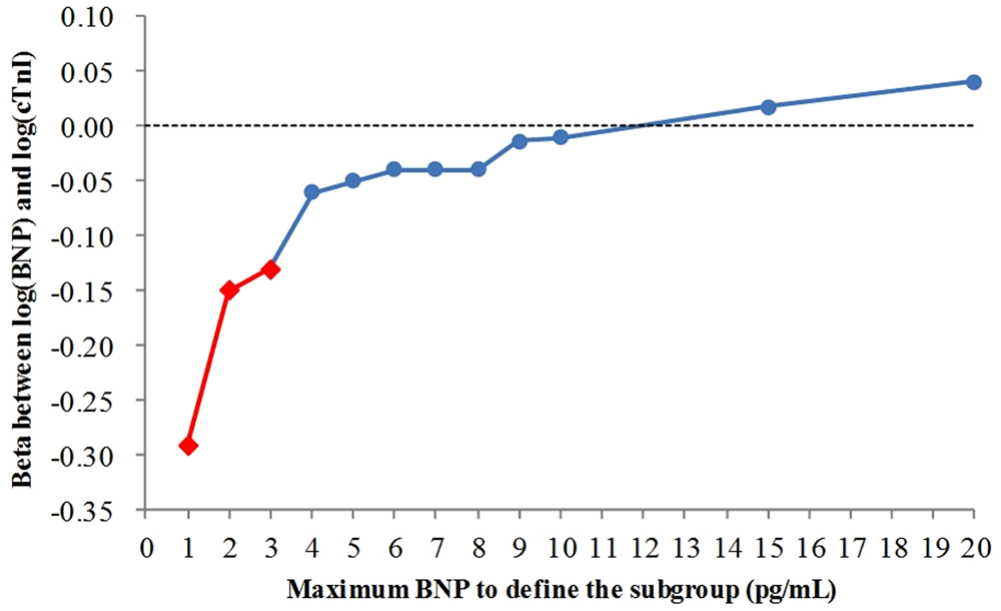
BNP cut-off levels to define sub-populations (X-axis) and beta values between log(BNP) and log(cTnI) (Y-axis). The red diamonds () signify beta values with p-values less than 0.005. The dotted line signifies beta = 0. BNP, B-type natriuretic peptide; cTnI, cardiac troponin I.

We performed covariance structure analyses with 12 subgroups of subjects stratified based on the upper limits of BNP levels at 1, 2, 3, 4, 5, 6, 7, 8, 9, 10, 15 or 20 pg/mL. The numbers of subjects in these subgroups were 81, 287, 516, 730, 918, 1,070, 1,205, 1,329, 1,416, 1,490, 1,747 and 1,860, respectively. We then performed covariance structure analyses to identify factors that are associated with elevated log(cTnI) within these subgroups and to determine beta values between log(BNP) and log(cTnI). The beta value between BNP and cTnI levels was negative (−0.29) in the subgroup with the BNP cut-off levels at 1 pg/mL (Fig. 3a, N = 81) but positive (0.04) in the subgroup with the BNP cut-off level at 20 pg/mL (Fig. 3b, N = 1,860). Figure 4 shows the plots of beta values of all the subgroups in the Y-axis against BNP cut-off levels in the X-axis. A sharp drop in the beta value was observed as the BNP cut-off level decreased from 20 pg/mL to 1 pg/mL. In particular, the beta values for the subgroups with BNP cut-off levels at 1, 2, and 3 pg/mL demonstrated strong significance (p < 0.005).

### Multivariate analyses

To evaluate the contribution of obesity to the elevation of cTnI level, we performed multivariate analyses for log(cTnI) for the total cohort and for a subgroup with BNP level lower than 4 pg/mL (Table 3). Similar to the results of the covariance structure analyses, the coefficient of log(BNP) against log(cTnI) in the subgroup with BNP level lower than 4 pg/mL was inversed (−0.423), whereas it was positive (0.149) in the total cohort. In the subgroup with BNP level lower than 4 pg/mL, BMI was not significantly associated with log(cTnI).

**Table 3:** Multivariate Analysis for Log(cTnI) for the Total Cohort and a Subgroup with BNP Level Lower than 4 pg/mL.

	Total Cohort				BNP Level Lower Than 4 pg/mL			
	Coefficient	SE	t	p-value	Coefficient	SE	t	p-value
Intercept	−2.698	0.221	−12.23	<0.001	−2.647	0.529	−5.01	<0.001
Log (BNP)	0.149	0.038	3.91	<0.001	−0.423	0.130	−3.24	0.001
Gender	0.281	0.036	7.77	<0.001	0.309	0.085	3.62	<0.001
Age	0.020	0.002	12.91	<0.001	0.021	0.004	5.69	<0.001
BMI	0.015	0.005	3.23	0.001	0.008	0.010	0.76	0.446
SBP	0.003	0.001	3.67	<0.001	0.005	0.002	2.03	0.043
Hb	0.015	0.013	1.13	0.260	0.015	0.032	0.46	0.647
eGFR	0.005	0.001	4.94	<0.001	0.005	0.002	2.35	0.019

BNP, B-type natriuretic peptide; cTnI, cardiac troponin I; BMI, body mass index; SBP, systolic blood pressure; Hb, haemoglobin; eGFR, estimated glomerular filtration rate; SE, standard error.

## Discussion

In this study, we confirmed that BNP was positively correlated with cTnI in the total cohort (Fig. 1) and that this positive correlation was maintained in the group with the BNP cut-off level at 20 pg/mL (Fig. 3b). We think that this is consistent with previous reports that showed the elevation of cTnI in subjects with chronic cardiac diseases, including congestive heart failure, left ventricle hypertrophy, and cardiomyopathy^8^, in which case BNP level is usually elevated, as well as the elevation of BNP in subjects with acute myocardial infarction^18^, in which case cTnI level is usually elevated.

Regarding the BNP assay that we employed in this study, 5.8 pg/mL was the lowest measurable BNP concentration according to the package insert. For the purpose of this study, however, we confirmed that the LoQ was 0.8 pg/mL by the definition described in Materials and Methods (Table 2). We then proceeded to define the subgroups with BNP cut-off levels as low as 1 pg/mL, which provided a basis for the breakthrough in elucidating the pathogenic role of low BNP level. By performing covariance structure analyses using subgroups with BNP cut-offs equal to or lower than 20 pg/mL, we confirmed that the beta values of log(BNP) against log(cTnI) decreased as the BNP cut-off levels decreased from 20 pg/mL to 1 pg/mL, and the inverse correlation became significant (p < 0.005) at BNP cut-off levels lower than 4 pg/mL (Fig. 4). From these results, we concluded that subjects with BNP levels lower than 4 pg/mL were susceptible to myocardial injury due to decreased BNP level. These results are consistent with the findings of our previous report that low BNP level was significantly associated with the incidence of IHD (p < 0.001)^16^ and support the hypothesis that insufficient BNP level may play a pathogenic role in the occurrence of cardiac disorders.

Notably, the association between BMI and log(cTnI) in the low-BNP group was not significant (Tables 3 and S1). Because increased BMI is reported to be associated with reduced BNP level^13–15^, the inverse association between cTnI and BNP may indicate that cTnI elevation did not directly result from reduced BNP level but rather from increased BMI, which coincidentally reduced BNP level. However, the results demonstrated that the elevation in log(cTnI) was inversely and directly associated with BNP but not with BMI in the low-BNP group (Tables 3 and S1), whereas the elevation in log(cTnI) was positively associated with BNP and BMI in the control group (Tables 3 and S2). This result is consistent with the result of our previous report that obesity contributes to the incidence of IHD via low BNP levels^16^.

Pleiotropic cardio-protective effects exerted by BNP have been reported by several researchers^19^. These effects include natriuresis, diuresis, vasodilation, lusitropy, lipolysis, weight loss and improved insulin resistance. As mentioned earlier, BNP level is reduced in obese individuals, but BNP also affects weight loss, indicating the presence of a balance between the effect of obesity to reduce BNP level and the effect of BNP to improve obesity. As we reported earlier, BNP level was also inversely associated with insulin resistance in patients with heart failure^20^. Therefore, a similar balance may exist between the effect of insulin resistance to reduce BNP level and the effect of BNP to improve insulin resistance. In this regard, a subject with low BNP level is at a status in which the effect of obesity or insulin resistance is dominant over the effect of BNP to improve obesity or insulin resistance. Under this circumstance, the cardio-protective effects of BNP, such as natriuresis, diuresis, vasodilation, or lusitropy, could be compromised, making the subject prone to myocardial injury.

In addition to the obesity and insulin resistance, genetic factors should also be taken into account as factors that reduce BNP levels. Polymorphism in the gene encoding BNP, natriuretic peptide precursor B (NPPB), has been reported to affect BNP expression levels^21^. As for polymorphisms in NPPB at T-381C, BNP level resulting from the genotype TT is lower than that resulting from the genotype TC or CC^21^. Seidelmann *et al*. reported that a polymorphism at the NPPB promoter, rs198389, was associated with the expression of NT-proBNP^22^. In that report, the authors showed that NT-proBNP expression, presumably BNP expression as well, was lower in subjects with the genotype AA at rs198389 than in subjects with the genotype AG or GG^22^. We, therefore, assume that low BNP levels result from a combination of factors including obesity, insulin resistance, and polymorphisms at NPPB and its promoter.

It is interesting that the slope of the curve shown in Fig. 4 looks biphasic; the slope is nearly horizontal when the BNP cut-off level is higher than 4 pg/mL, whereas the slope is nearly vertical at BNP cut-off levels lower than 4 pg/mL. This pattern may be reflective of a mechanism that compensates for the pathogenic effect of insufficient BNP level – a physiological mechanism that compensates for the compromised cardio-protective effect of the insufficient BNP level to attenuate injury to the cardiomyocytes, in a manner similar to the mechanism observed in the compensated status in patients with heart failure^23^.

Regarding the subject with a high cTnI level (826.1 pg/mL) who was excluded from this study as mentioned in Materials and Methods, the BNP level was only 1.9 pg/mL. This subject may also be another example of a case with cTnI elevation under low BNP conditions.

One of the reasons why it was possible for us to identify the pathogenic effect of low BNP level was that we performed the assessment in the general population. As mentioned earlier, it is known that BNP is expressed not only in patients with heart failure but also in patients with acute myocardial infarction^24^. Therefore, it would have been very difficult for us to identify subjects with BNP levels as low as those observed in the population in this study if we had chosen clinical patients with cardiac diseases as the study subjects. However, the putative mechanism by which a BNP level lower than that physiologically required exerts a pathogenic effect could be generalized to patients with cardiac diseases if, via the determination of influences of factors that suppress BNP expression (e.g., high BMI, insulin resistance, and genetic factors), we can assess whether BNP level in those patients are lower than that physiologically required. If this becomes possible, the monitoring of BNP level would be greatly beneficial for patients with cardiac diseases as well as for the general population.

### Limitations of the study

This study has the following limitations. Although the total number of subjects was high (2,001), the percentage of subjects with cTnI level greater than the 99^th^ percentile (26.2 pg/mL) was only 1.3% (27 subjects), and thus, the bias in the population may have affected the results. The other limitation is that the range of the parameters of health status in this population was comparatively narrow; for example; the BMI was within 20.6 and 24.8 in 50% of the population. Therefore, slight deviations from the overall population (e.g., subjects with extremely high BMI) may have affected the results. Last but not least, although the conclusion that extremely low BNP level is one of the causes for the development of cardiovascular diseases is consistent with previous reports (e.g., obesity is associated with higher IHD risk; low BNP is associated with the incidence of IHD; elevated cTnI level is associated with cardiovascular risks in the general population), we do not have prospective and direct evidence, which need to be obtained in the future.

## Conclusions

In subgroups with BNP levels lower than 4 pg/mL, elevation in cTnI level was inversely associated with BNP (p < 0.005), which suggests that insufficient BNP may play a pathogenic role in the occurrence of cardiovascular abnormalities.

## Methods

### Study population

We conducted this study at Takeda Hospital Medical Examination Center and Niigata Medical Association of Occupational Health. The study protocol conformed to the ethical guidelines of the 1975 Declaration of Helsinki and was approved by the Takeda Hospital Institutional Review Board and the Ethics Committee at Niigata Medical Association of Occupational Health. Upon obtaining informed consent, we recruited 2,005 subjects older than 38 years who visited Takeda Hospital Medical Examination Center or Niigata Medical Association of Occupational Health for their annual health check-up and showed no apparent symptoms of cardiac disease during doctor interviews. Among the recruited subjects, we excluded subjects with outlying laboratory test results by Dixon’s method^24^ and subjects with an eGFR below 30 mL/min/1.73 m^2^. This resulted in the exclusion of one subject who exhibited an extremely high cTnI level (826.1 pg/mL) and three subjects who exhibited eGFR below 30 mL/min/1.73 m^2^, corresponding to the enrolment of 2,001 subjects (884 females and 1,117 males) in total.

### Clinical and laboratory tests

All subjects underwent routine biochemical and haematological analyses for their health check-ups. BNP level was measured by an ARCHITECT BNP-JP assay (Abbott Laboratories, Abbott Park, IL, USA). According to the package insert of the BNP assay, the analytical sensitivity, which is defined as the concentration of the mean of the blank + 2 SD, was 5.8 pg/mL. cTnI level was measured with an ARCHITECT STAT High-Sensitivity troponin I assay (Abbott Laboratories, Abbott Park, IL, USA). According to the package insert of the cTnI assay, the 99^th^ percentile of cTnI level in an apparently healthy population was 26.2 pg/mL, the LoD was 1.9 pg/mL, and the CV% at the 99^th^ percentile was 4.0%. All blood samples were drawn from participants in a sitting position on the following morning after they had fasted overnight. BMIs were calculated based on the subjects’ heights and weights. The information regarding gender, age, medical history and smoking habits was collected from interviews. The estimated glomerular filtration rate (eGFR) was calculated using equations for the Japanese population, as reported previously^25^. The Framingham Risk Score was calculated according to a previously reported formula^17^.

Chronic kidney disease (CKD) was defined as present when a subject was under treatment for the disease, had an eGFR less than 60 mL/min/1.73 m^2^ or had positive urine protein results. Dyslipidaemia was defined as present when a subject was under treatment for the condition, had steatosis, had a high-density lipoprotein cholesterol (HDL-C) level lower than 40 mg/dL, had a low-density lipoprotein cholesterol (LDL-C) level higher than 139 mg/dL or had a triglyceride (TG) level higher than 149 mg/dL. Hypertension was defined as present when a subject was under treatment for the condition, had a systolic blood pressure (SBP) higher than 139 mmHg or had a diastolic blood pressure (DBP) higher than 89 mmHg. Diabetes was defined as present when a subject was under treatment for the disease, had a fasting blood glucose (FBG) level higher than 125 mg/dL or had an HbA1c value (NGSP) higher than 6.4%.

### Validation of the BNP assay

To assess the quantifiable range of the BNP assay solely for the purposes of this study, we performed a reproducibility test with serial dilutions of samples that were prepared from Control L and Calibrator A, which are components of the BNP assay. We prepared the serial dilution samples by serially diluting Control L (BNP = 54.2 pg/mL) with Calibrator A (BNP = 0.0 pg/mL) by twofold to BNP level below 1 pg/mL. The reproducibility test was performed by repeating the measurements ten times for each sample. We defined the LoQ as the lowest BNP concentration for which the error, which was taken as the sum of the CV% and absolute bias, was equal to or less than 30% based on reports in the literature^26^. The absolute bias was defined as the absolute value of the bias, which was the percentage of deviation of the measured value from the expected value.

### Metabolic derangement in the low BNP subgroups

To evaluate the presence of metabolic derangement in patients with extremely low BNP levels, we determined the percentage of subjects who exceeded the ULN WC or BMI. According to the report from the Japan Society for the Study of Obesity, 90 cm for women and 85 cm for men were used as the ULN for WC and 25 kg/m^2^ was used as the ULN for BMI^27^. Subgroups were obtained from the total cohort by limiting the maximum BNP level to 1, 2, 3, 4, 5, 6, 7, 8, 9, 10, 15 or 20 pg/mL.

### Statistical analyses

We used JMP 11.0.0 (SAS Institute Inc., Cary, NC, USA) for multivariate analyses. To analyse the factors that contributed to the elevation of cTnI level, we used IBM SPSS Amos (IBM, Armonk, New York, USA) to perform covariance structure analysis by fitting the data into structural equation models (SEM), with which we successfully elucidated the relationship among factors involved in cardiovascular pathogenesis^15,16,28–32^. For the analyses, we converted BNP into log(BNP) and cTnI into log(cTnI) because the distributions were non-Gaussian when confirmed by Shapiro-Wilk tests. For the purpose of conversion, we assigned cTnI values lower than 0.1 pg/mL to 0.05 pg/mL. For the assessment of the parameters that contributed to cTnI elevation, we chose log(BNP), gender, age, BMI, SBP, eGFR and Hb as the possible risk factors and categorized the rest of the factors into e1.

To assess the mechanism by which cTnI levels were associated with BNP levels in low BNP subgroups, we defined the subgroups among the overall population by limiting the maximum BNP level to cut-off values of 1, 2, 3, 4, 5, 6, 7, 8, 9, 10, 15 and 20 pg/mL and performed covariance structure analyses for each subgroup. P values of <0.05 were considered to indicate statistical significance.

### Data Availability

The datasets obtained during this study except for those attached as Supplementary Information are not publicly available due to privacy concerns.

## Electronic supplementary material

Supplementary Information
