# Supplementary material for: Increased Levels of Cardiac Troponin I in Subjects with Extremely Low B-type Natriuretic Peptide Levels
Source: Sci Rep. 2018 Mar 23;8:5120. doi: 10.1038/s41598-018-23441-z (PMC5865159; doi:10.1038/s41598-018-23441-z)
Supplement: Supplementary file 1 — Supplementary Information [file 41598_2018_23441_MOESM1_ESM.pdf]

Increased Levels of Cardiac Troponin I in Subjects with Extremely Low B-type Natriuretic Peptide Levels

Authors: Satoshi Sugawa[1*], Izuru Masuda[2], Kiminori Kato[3], Michihiro Yoshimura[4]

[1]Diagnostics Division, Abbott Japan Co., Ltd., Tokyo, Japan

[2]Takeda Hospital Medical Examination Center, Kyoto, Japan

[3]Niigata Medical Association of Occupational Health, Inc., Niigata, Japan

[4]Division of Cardiology, Department of Internal Medicine, The Jikei University School of Medicine, Tokyo, Japan

Table S1: Estimation with Structural Equation Model against Log(TnI) in the group B, a group of subjects with BNP values equal to or less than 1 pg/mL (N = 81).

Clinical Factor			Estimate	SE	Test statistic	p-value	Standard regression
	←	Gender	0.408	0.256	1.597	0.110	0.408
	←	Age	0.018	0.010	1.701	0.089	0.018
	←	eGFR	0.003	0.005	0.501	0.616	0.003
Log(cTnI)	←	SBP	0.011	0.007	1.568	0.117	0.011
	←	Log(BNP)	-1.186	0.436	-2.718	0.007	-1.186
	←	BMI	0.022	0.026	0.825	0.409	0.022
	←	Hb	-0.176	0.087	-2.032	0.042	-0.176

Table S2: Estimation with Structural Equation Model against Log(TnI) in the group B, a group of subjects with BNP values equal to or less than 20 pg/mL (N = 1,860).

Clinical Factor			Estimate	SE	Test statistic	p-value	Standard regression
Log(cTnI)	←	Gender	0.270	0.038	7.111	<0.001	0.270
	←	Age	0.021	0.002	12.549	<0.001	0.021
	←	eGFR	0.005	0.001	4.933	<0.001	0.005
	←	SBP	0.004	0.001	3.880	<0.001	0.004
	←	Log(BNP)	0.085	0.044	1.914	0.056	0.085
	←	BMI	0.012	0.005	2.470	0.014	0.012
	←	Hb	0.017	0.014	1.245	0.213	0.017
